# Non-contact lung disease classification via orthogonal frequency division multiplexing-based passive 6G integrated sensing and communication

**DOI:** 10.1038/s43856-025-01181-2

**Published:** 2026-01-06

**Authors:** Hasan Mujtaba Buttar, Muhammad Mahboob Ur Rahman, Muhammad Wasim Nawaz, Adnan Noor Mian, Adnan Zahid, Qammer H. Abbasi

**Affiliations:** 1 Electrical Engineering Department, Information Technology University, Lahore, Pakistan; 2 Computer Engineering Department, The University of Lahore, Lahore, Pakistan; 3 Computer Science Department, Information Technology University, Lahore, Pakistan; 4 School of Engineering and Physical Sciences, Heriot-Watt University, Edinburgh, UK; 5 James Watt School of Engineering, University of Glasgow, Glasgow, UK; 6 College of Engineering, Abu Dhabi University, Abu Dhabi, UAE

**Keywords:** Chronic obstructive pulmonary disease, Diagnostic markers

## Abstract

**Background:**

The screening tools for respiratory diseases typically involve spirometry (for asthma and COPD), CT scans (for interstitial lung disease), chest X-rays (for pneumonia and tuberculosis), and sputum analysis (for tuberculosis).

**Methods:**

This work examines a diagnostic approach whereby a subject’s chest is radio-exposed to non-ionizing 6G/WiFi multi-carrier radio signals at a frequency of 5.23 GHz. The fact that each respiratory disease modulates the amplitude, frequency, and phase of each radio frequency differently allows us to screen for five respiratory diseases: asthma, chronic obstructive pulmonary disease, interstitial lung disease, pneumonia, and tuberculosis. We collect a new dataset (OFDM-Breathe) from 220 individuals in a hospital setting, including 190 patients and 30 healthy controls. The dataset contains over 26,000 s of radio signal recordings across 64 frequencies. Several machine learning and deep learning models are evaluated to classify disease type based on the discriminatory signatures of radio signals.

**Results:**

We learn that a vanilla convolutional neural network achieves 98% accuracy in differentiating between the five respiratory diseases, along with strong performance in precision, recall, and F1-score. An ablation study demonstrates that reliable screening with up to 96% accuracy is possible using only eight frequencies, representing just 12.5% of the total bandwidth and leaving 87.5% available for 6G/WiFi data communication.

**Conclusions:**

The proposed method could enable real-time respiratory disease screening, could help realize the health equity in developing countries, and lays the groundwork for 6G/WiFi-enabled integrated sensing and communication platforms for healthcare systems of the future.

## Plain language summary

This study explores a non-ionizing, contactless screening method for five prevalent lung diseases using 6G/WiFi radio signals (to complement the conventional methods such as chest X-rays, spirometry, CT scans). It leverages the fact that each lung disease leads to an abnormal breathing pattern which in turn alters the reflected signals in a unique way. Data from 220 individuals (both healthy and patients) were collected in a clinical setting, and AI models were trained to distinguish between disease-specific patterns. The approach demonstrated high accuracy in detecting respiratory conditions and holds promise as a low-cost diagnostic tool, especially in remote or resource-limited settings (such as in developing countries). The findings also highlight the potential of 6G/WiFi signals for broader health diagnostics.

## Abstract (web summary)

Buttar et al. explore a contactless method for screening five respiratory diseases using 6G Integrated Sensing And Communication Orthogonal Frequency Division Multiplexing (ISAC OFDM) signals from software-defined radios. Using deep learning, the approach enables accurate, real-time screening for Asthma, Chronic Obstructive Pulmonary Disease, Interstitial Lung Disease, Pneumonia and Tuberculosis.

## Introduction

Respiratory diseases are caused by a number of risk factors, e.g., environmental factors (i.e., air pollution), non-healthy life-style (e.g., tobacco smoking), long-term exposure to allergens (e.g., irritating particles or gases, workplace fumes), bacterial infections, physical exertion, stress, and genetic factors. Respiratory diseases are a critical global health concern, and put a significant burden on healthcare sector in lieu of health insurance costs worldwide. The world health organization (WHO), in its 2019 report, identified respiratory diseases as the third-largest cause of mortality globally, behind cardiovascular diseases and cancer, with approximately 3.8 million annual deaths attributed to them^1^.

Respiratory diseases fall into two distinct categories: obstructive and restrictive lung diseases, each significantly impacting lung function and breathing capacity in unique ways. Obstructive lung diseases involve a narrowing or obstruction of the airways, impeding the proper flow of air into and out of the lungs. This primarily affects exhalation, resulting in airflow limitations^2^. In contrast, restrictive lung diseases involve stiffness or damage to the lung tissue itself, impairing lung expansion and reducing lung volume.

Conventional lung disease screening methods, such as chest X-rays, computed tomography (CT) scans, and spirometry, remain essential diagnostic tools but present notable drawbacks. These include exposure to ionizing radiation, the requirement for physical contact, which may be sometimes undesirable in post-COVID-19 context, high operational costs, and dependence on trained personnel and clinical infrastructure. Consequently, there is an increasing demand for alternative screening techniques that are non-invasive, contactless, and scalable. Such approaches could complement or, in specific scenarios, serve as practical substitutes for traditional diagnostics, particularly in remote or resource-constrained settings.

During last two decades, researchers have studied various non-invasive and non-contact lung disease classification methods that range from medical imaging (X-ray, CT scan)-based methods, to acoustics (cough, breathing sound, lung sound)-based methods, to radio signals (radar, software-defined radios)-based methods. A vast majority of these works rely upon a range of artificial intelligence (AI) techniques in order to do rapid COVID-19 and lung cancer detection in particular, and lung disease classification in general (see the survey articles^3–5^ and references therein). There also exist methods such as chemical analysis of exhaled breath^6^, etc. The literature on lung disease screening is extensive; therefore, we have organized the related works below based on the sensing modality used.

The medical imaging-based methods utilize data such as chest CT scans, chest X-ray images, etc., and traditionally require the expertise of a radiologist or a physician. However, the landscape has drastically changed recently, whereby AI techniques have shown lots of promise for automatic respiratory disease classification using medical imaging data^7^. In fact, automatic COVID-19 disease diagnosis by doing AI-based analytics on various medical imaging modalities has been one of the most researched problems in the post-COVID-19 era^8^. Among such works, a fair amount of works investigate the feasibility of COVID-19 detection and diagnosis of other lung diseases using chest X-rays images only, owing to the fact that it is a less expensive, faster, and widely available technology^9,10^. Moving forward, we note that though the related work on lung disease diagnosis using medical imaging data is abundant, nevertheless, we discuss only a few related works below, due to the space constraints. Authors of^11^ acquire 412 chest X-ray images of Pneumonia patients, segments them to obtain various lung regions in order to extract eight statistical features, and achieves a remarkable accuracy of 95.39% using the multi-layer perceptron (MLP) classifier.^12^ utilizes chest CT scan images of 52 Pneumonia patients, extracts six features, utilizes a convolutional neural network (CNN), and reports an accuracy of 97%.^13^ considers a multi-clas classification problem whereby the aim is to differentiate between COVID-19, Pneumothorax, Pneumonia, lung cancer, and Tuberculosis. To this end, they apply fuzzy tree transformation to the X-ray images, do feature extraction, and implement three CNN-based pre-trained models, i.e., VGG-19, ResNet-50, and Inception v3, which achieve an accuracy of 95.61%, 96.15%, and 95.16%, respectively.^14^ considers a small dataset consisting of chest X-ray images of COVID-19 and Pneumonia patients, applies the fuzzy tree transformation to the images, do feature extraction using a multikernel local binary pattern, and implements traditional machine learning classifiers, which achieved an accuracy of 97.01%.^15^ considers a dataset comprising 3345 chest X-ray images of Pneumonia patients, implements a CNN that achieves an accuracy of 97.64%, and develops a PneumoniaApp with the hope that it helps physicians localizing the regions of lung opacity.^16^ enhances the quality of CT scan and X-ray images using k-symbol Lerch transcendent functions. Subsequently, two pre-trained deep learning models, AlexNet and VGG16Net, were developed to distinguish between three different lung conditions: pneumonia, COVID-19, and a healthy lung. Following image enhancement, the models achieved outstanding accuracies of 98.60% and 98.50% for the X-Ray and CT scan datasets, respectively. While the researchers have largely remained focused on early computer-aided detection of COVID-19, lung cancer, and Pneumonia using medical imaging data, we note that the research on AI-empowered diagnosis of Asthma, Chronic obstructive pulmonary disease (COPD)^17^, Interstitial lung disease (ILD)^18^, and Tuberculosis (TB)^19^ using CT scan and X-ray images is also picking up lately.

The Respiratory sounds-based methods utilize the fact that lung sounds exhibit significant changes due to different respiratory disorders. For example, Asthma causes wheezing that results from heavy mucus production or airway inflammation. Wheezing sounds are typically heard during expiration, but sometimes during inspiration. Wheezing and other such abnormal sounds could be heard when a sick person breathes or speaks. This has motivated the researchers to design a collection of methods that utilize one of the following modalities to diagnose COVID-19 and other lung diseases: the lung sounds (through auscultation), speech signals, breathing sounds, and cough sounds^20,21^. Below, we discuss the three acoustic methods, one by one.

The lung auscultation method measures lung sounds using an electronic stethoscope.^22^ does lung auscultation to record wheezing and crackle sounds of 126 pulmonary patients in a hospital setting, and implement an improved VGG network by incorporating a lightweight attention mechanism to diagnose various lung diseases (i.e., Asthma, COPD, ILD, Bronchiectasis, and Pneumonia).^23^ utilizes the ICBHI2017 challenge lungs dataset, extracts Mel-Frequency Cepstral Coefficients (MFCC) features, and implements a dual-channel CNN-plus-long short term memory (LSTM) for diagnosis of various respiratory diseases (i.e., COPD, Pneumonia, Bronchiectasis, Upper Respiratory Tract Infections and Bronchiolitis).

The voice-based method involves capturing high-fidelity speech recordings via a microphone.^24^ argues that laryngeal diseases could be diagnosed based on the noticeable change in vocal tracts that leads to a pathological voice.^25^ utilizes CNN and recurrent neural network (RNN) model for breathing event detection while during conversation, with the aim to detect COPD, Asthma, and general respiratory infections.

The cough-based method records high-fidelity cough sounds using smartphones or microphones. Cough-based diagnosis of various respiratory diseases, such as Asthma, COPD, Tuberculosis, Bronchitis, etc., through signal processing and AI-based analytics has attracted considerable attention recently^26–28^. acquires Pneumonia coughs, Asthma coughs, and Bronchitis coughs of children through microphones, and performs time-series statistical analysis, and time-frequency analysis in order to reliably detect childhood Pneumonia.^29^ applies spectral analysis, time-series analysis, and statistical methods with the aim to differentiate between the wet and dry coughs of children.^30^ does frequency-domain analysis of the cough of Asthmatic patients and concludes that it is distinct from the cough of non-Asthmatic patients as it has higher energy in low-frequency components. Finally,^31,32^ implements a CNN and RNN model in order to do COVID-19 detection based on cough.

Next, RF-based methods that utilize the radar and software-defined radio (SDR) are discussed below, one by one.

The radar-based methods utilize the classical range-Doppler principles in order to localize and track the objects of interest, i.e., rhythmic movement of human chest in case of (pulmonary and cardiac) health sensing^33,34^. In^35^, Li et. al. do joint estimation of the position of a person and his/her breathing rate in a non-line-of-sight scenario using a 77-GHz frequency-modulated continuous-wave (FMCW) radar.^36^ presents an adaptive variational mode decomposition algorithm for heartbeat signal extraction from FMCW radar data, amid artifacts such as random body movements, respiration, and their harmonics.^37^ utilizes the millimeter-wave radar—operating in the frequency range of (60−64) GHz—for heartbeat classification, distinguishing between normal sinus rhythm, bradycardia, and tachycardia. They detect normal sinus rhythm, bradycardia, and tachycardia with an accuracy of 85.9%, 92.2%, and 93.9%, respectively.^38^ performs a diagnostic cross-sectional study on eight postoperative patients, whereby a radar system is used to measure their respiratory rate during mechanical ventilation during spontaneous breathing. Authors of^39^ design an ultrasonic-based radar with desirable characteristics (e.g., highly linear, phase-canceling, self-injection-locked) which could reliably and simultaneously measure both the breathing rate and the heart rate of a person.^40^ elegantly monitors the delicate breathing signals of premature infants within the neonatal intensive care unit (NICU) using a sophisticated radar system operating in the 24-GHz ISM (industrial, scientific, and medical) band. Authors of^41^ aim to monitor the performance of cardiac system, whereby they radio-expose the human chest with radar signals and utilize generative AI methods to construct a seismocardiograph signal. Going beyond academic research,^42^ reports clinical validation of their Sleepiz One+ device, which utilizes a continuous-wave Doppler radar, for assessing respiratory performance of patients with COPD and Asthma. When compared to the thoracic effort belt, Sleepiz One+ demonstrates an exceptional accuracy of 99.5%, highlighting its potential for reliable, contactless respiratory monitoring.

The SDR-based health sensing methods monitor the human respiratory system based on small chest movements that are recorded through WiFi radio signals reflected off the human chest. In refs. ^43,44^, authors utilize the channel frequency response (CFR) data acquired-off the human chest in order to detect and classify three breathing patterns: normal, shallow, and elevated breathing.^45^ extends this further by classifying up to eight breathing patterns, i.e., eupnea, bradypnea, tachypnea, Biot, sighing, Kussmaul, Cheyne-Stokes, and central sleep apnea. Kawish et al.^46^ adopt a different approach where instead of the chest, they radio-expose the hand in order to differentiate between three kinds of breathing patterns, i.e., Tachypnea, Bradypnea, and Eupnea.^47^ utilizes SDR WiFi signals to screen people for COVID-19 by searching for irregular breathing patterns through their chest movement analysis. In^48^, Guan et al. employ a modified variational mode decomposition technique to extract respiratory and heartbeat signals from SDR WiFi signals. Their approach also enables accurate heart rate variability (HRV) measurement within 0.5 m range.^49^ aims to synthesize a photoplethysmography (PPG) signal using generative AI methods by utilizing the modulated orthogonal frequency division multiplexing (OFDM) signal reflected-off the chest of the chest of a healthy subject. Finally,^50^ radio-observes the chest of a subject in order to infer his/her hydration status in a non-contact manner.

There exist some other non-contact sensing modalities as well that could aid in diagnosis of pulmonary diseases. For example, exhaled breath analysis methods assess the chemical composition of exhaled breath using sensors and spectrometry, and can detect biomarkers for diseases like asthma, COPD, and lung cancer^6^. Another method is imaging with infrared thermography, which could assess respiratory function by detecting temperature variations related to breathing patterns and nasal flow. Pulse oximetry is yet another non-invasive method that measures Oxygen saturation in the blood and can be a quick indicator of respiratory function, though not specific for diagnosis^51^.

This work addresses a key research gap by demonstrating the use of wideband 6G/WiFi non-ionizing OFDM microwave signals for non-contact classification of five lung diseases (Asthma, COPD, ILD, Pneumonia, and TB) in diagnosed patients. Unlike prior studies, it leverages frequency, phase, and amplitude-modulated chest movement reflections, captured via OFDM sub-carriers, and transformed into CFR for AI-based disease identification. The proposed method introduces the OFDM-Breathe dataset, comprising 26,760 s of RF data at 64 microwave frequencies collected from 220 individuals (190 patients, 30 healthy) in a clinical setting. Deep learning models, particularly CNNs, achieved 98% test accuracy, while LSTM and transformer models reached 97%, and MLP-based models exceeded 88.8%. An ablation study showed that only 8 sub-carriers (12.5% of the bandwidth) are sufficient for 96% accurate classification, enabling efficient 6G integrated sensing and communication (ISAC) technique for health diagnostics. The approach advances non-invasive screening and supports emerging applications in smart homes, smart cities, and digital twin technologies.

Since this work utilizes radio sensing modality that captures the changes in the breathing pattern to differentiate between various diseases, we consider it imperative to first do a brief discussion about the pathophysiology of each of these five lung diseases from the perspective of how it impacts the breathing pattern.

A healthy respiratory system is typically marked by normal respiratory rate, deep breathing, and relaxed chest wall movement. Pulmonary diseases, however, alter this physiology in characteristic ways. Asthma, an obstructive disease affecting over 235 million people worldwide—particularly children (14%)—is marked by airway inflammation and constriction, producing wheezing, dyspnea, and accessory muscle use^52^. COPD, a progressive condition comprising chronic bronchitis and emphysema, affects 11.7% of the global population and causes around 3 million deaths annually, with patients exhibiting dyspnea, tachypnea, pursed-lip breathing, and barrel chest^1,53^. ILD, which leads to lung scarring and impaired oxygen transfer, affects over 4.7 million people worldwide, presenting with tachypnea, shallow breathing, and accessory muscle use^54^.

Infectious diseases further exacerbate the global burden of respiratory illness. Pneumonia (PN), a leading cause of morbidity and mortality across age groups, reduces lung expansion through inflammation and fluid accumulation, resulting in tachypnea, shallow breathing, and dyspnea^55^. TB continues to affect around 10.4 million individuals annually, with 1.4 million deaths reported in 2015, and is associated with cough, dyspnea, chest pain, wheezing, crackles, and hemoptysis^56^. These diverse disease mechanisms and breathing pattern abnormalities underscore the importance of developing reliable, non-invasive diagnostic tools for early detection and monitoring of pulmonary disorders.

Respiratory illnesses are treatable with early detection, yet variations in diagnostic standards contribute to high mortality rates, as diagnosis often relies on the expertise of clinicians, leaving room for misdiagnosis and irreversible harm. The current gold standard for lung disease diagnosis is multi-pronged and involves several sensing modalities. Clinical assessment through patient history and physical examination provides the foundation, while stethoscope-based auscultation identifies characteristic sounds such as wheezes and crackles in conditions like asthma and COPD. Imaging techniques, including chest X-rays and CT scans, remain indispensable for detecting pneumonia, tuberculosis, and lung cancer. Pulmonary function tests, particularly spirometry, quantify lung capacity and airflow for accurate diagnosis of obstructive airway diseases. Laboratory tests, including blood biomarkers and sputum cultures, detect infections and inflammatory conditions, while emerging studies explore microbial extracellular vesicles for disease classification using machine learning. Endoscopic procedures such as bronchoscopy further allow direct airway visualization and biopsy for histopathological confirmation.

Despite their clinical value, these traditional diagnostic methods are often invasive, time-consuming, costly, and unsuitable for daily screening, with modalities like CT scans raising additional concerns of radiation exposure. This underscores the urgent need for low-cost, non-invasive, and non-contact alternatives capable of enabling rapid, routine, and large-scale screening for early detection of pulmonary diseases. Addressing this gap is precisely the scope of the present work.

## Methods

### The experimental setup

**Fig. 1:**
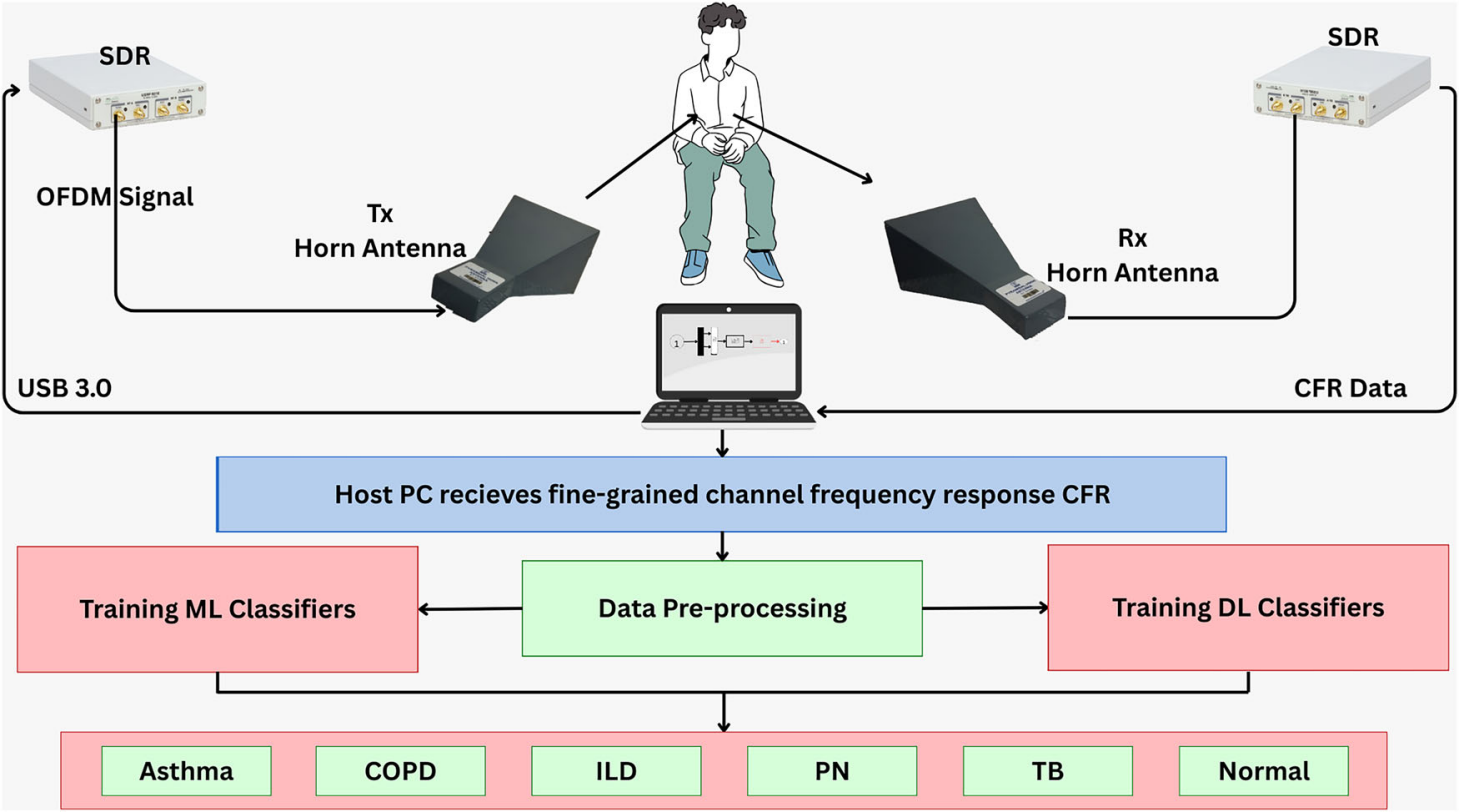
The proposed method. A software-defined radio (SDR)-based orthogonal frequency division multiplexing (OFDM) transceiver illuminates the chest of a subject with radio waves, collects the reflected signals, and passes them to various machine and deep learning methods, which ultimately classify a subject either as healthy or sick with a pulmonary disease.

The experimental setup for non-contact monitoring and identification of pulmonary diseases consists of two SDRs, basically, Universal Software Radio Peripheral (USRP) model B210 SDRs (see Fig. 1). MATLAB R2021a is used to program both the transmit and receive USRP SDRs. The two radios communicate by exchanging OFDM signals between them. Some pertinent details of the transmit USRP SDR and the receive USRP SDR are as follows.

*USRP SDR-based OFDM Transmitter*: The data is grouped in batches of 128 bits, and map them to quadrature phase-shift keying (QPSK) symbols. Then, 64-point inverse fast Fourier transform (IFFT) of the vector containing QPSK symbols is taken, and a cyclic prefix (CP) of 16 samples is appended, which sets the length of the OFDM frame to 80 samples. Finally, a 15 dBi antenna is utilized to transmit the OFDM signal.

*USRP SDR-based OFDM Receiver*: Having received an OFDM frame, first of all the CP is removed, followed by 64-point fast Fourier transform (FFT). Next, the channel gain *h*_*i*_ on *i*th sub-carrier is computed as follows: ${h}_{i}=\frac{{y}_{i}}{{x}_{i}}$, where *x*_*i*_,*y*_*i*_ is the sent and received QPSK symbol on *i*th sub-carrier, respectively. Note that ${x}_{i},{y}_{i},{h}_{i}\in {\mathbb{C}}$. The channel frequency response ${{{\bf{h}}}}={[{h}_{1},\cdots ,{h}_{N}]}^{T}$ constitutes the raw RF data, which is later fed to the machine learning (ML) and deep learning (DL) algorithms in order to classify each subject as either healthy or sick with a pulmonary disease.

**Fig. 2:**
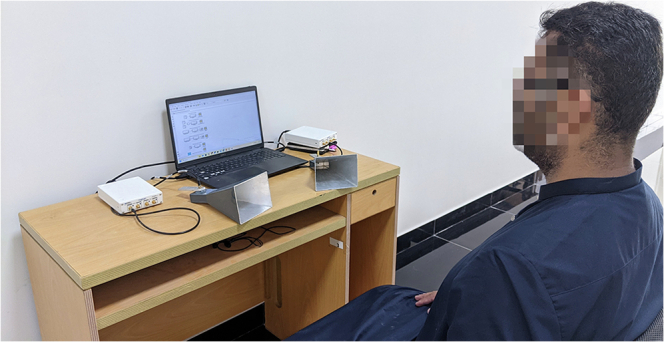
The experimental setup used for acquisition of the orthogonal frequency division multiplexing (OFDM)-Breathe dataset. It consists of two universal software radio peripheral (USRP) B210 software-defined radios (SDRs), two directional horn antennas, and a personal computer (PC).

Figure 2 provides a pictorial overview of the methodology of this paper, and Table 1 summarizes the values of the key parameters that have been used to configure the transmit and receive USRP SDRs.

**Table 1:** Important parameters for the software-defined radio (SDR) WiFi orthogonal frequency division multiplexing (OFDM) transceiver used for non-contact identification of lung diseases

Parameter	Type/Value
Modulation scheme	QPSK
No. of OFDM subcarriers	64
Size of FFT/IFFT	64 points
Size of cyclic prefix	16
Sampling rate	1000 samples/sec
Centre frequency	5.23 GHz
Gain (at Tx and Rx)	40 dB

This way, we acquired OFDM-Breathe dataset, first of its kind, which consists of 26,760 s of raw RF data at 64 distinct microwave OFDM frequencies, acquired from a total of 220 subjects in a hospital setting (30 healthy control subjects, and 190 pulmonary patients). Among the 190 patients, 45 have Asthma, 43 have COPD, 41 have TB, 30 have ILD, and 31 have PN.

### Data pre-processing

The following pre-processing steps are done in order to condition and prepare the recorded CFR data for the machine and deep learning models:

*Decimation-in-time*: The rhythmic inhalation and exhalation activity of the lungs (i.e., 12–20 breaths/min under sinus conditions, and 20–30 breaths/min under pathological conditions) implies that the frequency band of interest lies in a narrow range of 0–1 Hz. This allows us to do decimation-in-time (DiT) by a factor of 10 in order to reduce the sampling rate of the CFR data to 100 Hz. The DiT step reduces size of the OFDM-Breathe dataset by 10, while preserving essential respiratory features, thus alleviating the computational burden on ML and DL classifiers during the inference stage.

*Decimation-in-frequency*: Further, we have empirically observed that the human chest responds to the two adjacent OFDM sub-carriers *i*, *j* in a similar way, i.e., ∣*h*_*i*_ − *h*_*j*_∣ < *δ*_*m*_ and *∠*(*h*_*i*_ − *h*_*j*_) < *δ*_*p*_,  ∀ *i*, *j* = 1, . . . , *N* = 64 where *δ*_*m*_, *δ*_*p*_ are arbitrarily small numbers. This allows us to do decimation-in-frequency (DiF) by a factor of 4. The DiF step further reduces the size of the OFDM-Breathe dataset by 4, while retaining critical respiratory motion characteristics. This in turn helps reduce the computational and memory demands of the ML and DL classifiers at the later stage.

*Data segmentation:* Next, we do data segmentation, whereby we split the CFR data corresponding to each recording session of duration 30 s into three smaller segments of duration 10 s each. Thus, size of each segment is: ${{{\bf{H}}}}\in {{\mathbb{C}}}^{16\times 1000}$. Further, we repeat the class label across all three segments of that recording. This way, the size of the OFDM-Breathe dataset (in terms of number of examples/data points) increases by threefold. Specifically, the augmented dataset consists of 540 labeled examples for Asthma, 516 for COPD, 492 for TB, 372 for PN, and 360 labeled examples for each ILD and normal classes. Segmentation also enhances the model generalizability and allows the ML and DL models to do finer temporal analysis of breathing patterns.

*Denoising*: We utilise the 8-level discrete Wavelet transform with db4 mother wavelet to denoise (low-pass filter) the CFR data, and to remove the artifacts. This step enhances the signal quality by preserving physiologically relevant breathing patterns while removing extraneous variations due to motion-induced artifacts and high-frequency noise.

*Data reshaping*: Finally, we reshape the denoised CFR data as per needs of various ML and DL classifiers. Reshaping ensures compatibility with different model architectures, enabling effective feature learning and classification.

### Machine learning classifiers

We use Matlab in order to feed the OFDM-Breathe dataset to the following ML classifiers and their variants: linear discriminant (LD), K-nearest neighbours (KNN), support vector machine (SVM), and ensemble classifier. Furthermore, we also train and evaluate three variants of MLP, i.e., narrow, medium, and wide MLP with a single hidden layer, but with 10, 25, 100 neurons, respectively. We also implement bi-layered MLP (tri-layered MLP), which has two (three) dense layers with 10 neurons in each layer.

*Evaluation strategy for the DL models*: In order to do a fair evaluation of the performance of ML and MLP models, we created a balanced dataset by considering 30 subjects from each disease class (as well as from the normal healthy control class) from the OFDM-Breathe dataset. This means we utilize the data of a total of 180 subjects, corresponding to the six classes. We test our ML models on *unsegmented data*, which means we have a total of 180 data points corresponding to 180 subjects, which is, in turn, equivalent to doing *individual-dependent testing*. This evaluation methodology avoids data leakage issue, i.e., the data of each subject is either included in the training set or the test set, but not both. Finally, due to the small size of (unsegmented) OFDM-Breathe dataset, we use K-fold (with *K* = 5) cross-validation strategy in order to avoid the over-fitting issues.

### Deep learning classifiers

We implement three DL models, namely, Transformer, LSTM, and CNN, in Python, Keras, and Tensorflow framework.

#### The transformer model

The Vanilla Transformer model that we have implemented consists of an embedding layer, followed by a Transformer block, followed by pooling, dropout, and dense layers. Together, all these layers help Transformer model learn the underlying representations of input RF sequences and perform the classification task at hand. Below, we provide a compact briefing about each layer of our Transformer model:

*Embedding layer*: The input sequence goes through two separate embedding layers, one for tokens (which gives us a dense, fixed-length embedding vector for each token) and another for token indices, i.e., positions (which helps the model to learn the relative positions of tokens in the input sequence). Eventually, we combine token embeddings with positional embeddings. This layer is parameterized by the following: (1) *max-len*, which is maximum length of input sequences, (2) *embed-dim*, which specifies the size of the embedding vector for each token in the input sequence, (3) *vocab-size*, which specifies the size of the vocabulary, i.e., the total number of unique tokens in the input data.

*Transformer block*: The Transformer block consists of three main components: (1) Query, Key, and Value vectors, (2) Attention scores, (3) Weighted sum of values. Specifically, each input token (or position) is first associated with three vectors: query, key, and value. Then, the self-attention mechanism computes attention scores between each query and key pair. Finally, the attention scores are used to compute a weighted sum of the corresponding value vectors. This weighted sum represents the attended information, where tokens with higher attention scores contribute more to the final output. The weighted sum operation is akin to focusing on specific parts of the input sequence based on their relevance to the current context. All in all, by incorporating self-attention mechanisms, our Transformer model can capture long-range dependencies in the input RF sequences more effectively. The Transformer block is parameterized by the following: (1) *embed-dim*, which is the dimension of the embedding vectors, (2) *num-heads*, which represents the number of attention heads, (3) *ff-dim*, which specifies the size of the hidden layer in the feedforward network inside the Transformer block.

*Global average pooling layer*: Next, we perform global average pooling across the time dimension (sequence length) of the input sequence, in order to reduce the spatial dimensions of the input sequence to a single vector, aggregating information from all tokens.

*Dropout and dense layers*: Next, we apply dropout regularization with a dropout rate of 0.1 to prevent overfitting by randomly setting a fraction of input units to zero during training. The dropout layers are interlaced between two dense layers with 20 units and 6 units, respectively.

Table 2 provides a compact summary of the architecture of the Transformer model that we have implemented.

**Table 2:** Architecture of the vanilla Transformer model

Layer type	Output shape	Parameters
Input layer	(None, 16)	0
Token and position embedding	(None, 16, 32)	640,416
Transformer block	(None, 16, 32)	19,040
Global avg. pooling	(None, 32)	0
Dropout	(None, 32)	0
Dense	(None, 20)	660
Dropout	(None, 20)	0
Dense	(None, 6)	126

*Hyperparameters*: We set *embed-dim* = 32, *num-heads* = 4, and *ff-dim* = 32.

#### The LSTM model

We tried various configurations of the Vanilla LSTM model. It turns out that the LSTM that gives the best performance is a relatively shallow LSTM with an input layer, two LSTM layers (with 64,16 units, respectively), each followed by a dropout layer with a dropout ratio of 0.2, followed by one dense layer of size 6. We use Softmax activation function at the final layer.

*Hyperparameters:* We use categorical focal crossentropy (with *γ* = 1) as loss function, we use Adam optimizer. We do 100 epochs, and set the batch size to 8192.

#### The CNN model

We tried various configurations of the Vanilla CNN model. It turns out that the CNN that gives the best performance is a relatively shallow CNN with three convolutional (CNV) layers (with 64,32,16 filters respectively), one flatten layer, and three fully connected (FC) layers (of size 64,32,6 respectively). Further, the following holds for each CNV layer: kernel size = 3, padding = same, stride = 1. Note that each CNV layer is followed by a max pooling layer with pool size = 2, while each FC layer is followed by a dropout layer with a dropout ratio of 0.3. We use ReLU activation function at each layer, expect the last one, where Softmax is used.

*Hyperparameters:* We use categorical focal crossentropy (with *γ* = 1) as the loss function. We use Adam optimizer, we do 100 epochs, and set the batch size to 8192.

*Evaluation strategy for the DL models*: Keeping in mind that all three DL models are data hungry due to a large number of trainable parameters, we utilize segmented version of OFDM-Breathe dataset in order to train and evaluate all three DL models. Further, we utilize a train-validation-test ratio of 70-15-15- for all three DL models. Finally, we utilize the full OFDM-Breathe dataset (which is slightly imbalanced) for training and evaluation of the three DL models.

### Ethical approval

This study was approved by the ethical institutional review board (EIRB) of Information Technology University (ITU), Lahore, Pakistan, as well as from the EIRB of the local hospital in Lahore. Each subject (healthy or patient) and/or their representative read and signed the informed consent form. Finally, as per approved protocol, we only collected data from those pulmonary patients who were in clinically stable condition.

### Reporting summary

Further information on research design is available in the Nature Portfolio Reporting Summary linked to this article.

## Results

The M.L., M.L.P. and D.L. models were implemented on a workstation with the following specs: Intel Core i7 3.6 GHz processor with 32 GB RAM.

### Performance metrics

The following performance metrics were utilized to assess the performance of our ML and DL classifiers: accuracy, precision, recall, F1-score, support, true negative rate, false positive rate, false negative rate, Matthews correlation coefficient, Jaccard index, confusion matrices, and accuracy/loss vs. epochs curves. Next, we provide a compact definition of each of the metrics utilized in this work. ${{{\rm{Accuracy}}}}=\frac{{{{\rm{Correct}}}}\,{{{\rm{predictions}}}}}{{{{\rm{Total}}}}\,{{{\rm{observations}}}}}\times 100=\frac{TN+TP}{TN+TP+FN+FP}\times 100$. Here, *TN* represents a true negative, *TP* represents a true positive, *FN* represents a false negative, and *FP* represents a false positive. Precision measures the proportion of correctly predicted positive instances out of all instances predicted as positive. It is defined as: Precision $P=\frac{TP}{TP+FP}$. Recall measures the proportion of correctly predicted positive instances out of all actual positive instances. It is defined as: Recall $R=\frac{TP}{TP+FN}$. The F1-score is the harmonic mean of precision and recall, providing a balanced measure of the model’s performance. It is defined as: F1-score =$\frac{2\times (P\times R)}{P+R}$. Support refers to the number of actual occurrences of each class in the dataset. The true negative rate (TNR), also known as specificity, measures the proportion of actual negatives correctly identified by the model: $TNR=\frac{TN}{TN+FP}$. The false positive rate (FPR) quantifies the proportion of actual negative cases that are incorrectly classified as positive: $FPR=\frac{FP}{FP+TN}=1-TNR$. The false negative rate (FNR), also called the miss rate, represents the proportion of actual positive cases that are incorrectly classified as negative: $FNR=\frac{FN}{FN+TP}=1-TPR$. The Matthews correlation coefficient (MCC) is defined as: $MCC=\frac{(TP\times TN)-(FP\times FN)}{\sqrt{(TP+FP)(TP+FN)(TN+FP)(TN+FN)}}$. MCC is a balanced metric, even for imbalanced datasets. It ranges from −1 (total misclassification) to +1 (perfect classification), with 0 indicating random guessing. The Jaccard index (JI), also called intersection over union, measures the similarity between the predicted and actual positive classes: $JI=\frac{TP}{TP+FP+FN}$. JI ranges from 0 (no overlap) to 1 (perfect overlap). In addition, we also do a performance comparison of the various ML and DL classifiers by means of confusion matrices. Furthermore, we also do latency analysis of our ML and MLP classifiers in terms of training time (seconds) and prediction speed (observations/sec). Finally, we also provide the progression of accuracy and loss functions against the number of epochs for our DL models.

### Performance of the ML, MLP, and DL models

Table 3 summarizes the accuracy reported by various ML and MLP models. We observe that the MLP model, along with its variants, reports an overall accuracy that is  > 88.8% and thus outperforms all the ML models. This is followed by the SVM (quadratic), which reports an accuracy of 85.7% and SVM (cubic kernel) with accuracy of 84.7%. Note that the K-nearest neighbors model, linear discriminant model, ensemble method all registered an intermediate performance, and thus, their results are omitted for the sake of brevity. Additionally, Table 3 also outlines the prediction speed and training time of all the ML and MLP classifiers. The MLP and its variants distinguish themselves by achieving the highest prediction speeds among all machine learning classifiers, while their training time is comparable with all other ML classifiers.

**Table 3:** Accuracy, prediction speed and training time of various machine learning (ML) and multi-layer perceptron (MLP) models

ML algorithms	Accuracy (%)	Prediction speed (obs/sec)	Training time (sec)
SVM (Quadratic)	85.7	4.3	12374
SVM (Cubic)	84.7	5.6	9864.2
MLP (Narrow)	93.2	48	8670.8
MLP (Medium)	95.9	52	4365
MLP (Wide)	95.3	65	7240.1
MLP (Bilayered)	92.5	56	6347.5
MLP (Trilayered)	88.8	63	6643.2

Table 4 summarizes the precision, recall F1-score, TNR, FPR, and FNR for each of the six classes obtained by a number of ML classifiers, as well as the MLP classifier. We make the following observations. The SVM (quadratic and cubic) models perform well overall, especially for COPD, but struggle with TB due to high FNR. The MLP (Narrow) model shows poor recall and high FNR for several classes, particularly ILD and TB, indicating a tendency to miss positive cases. MLP (Medium, Wide, Bilayered, and Trilayered) models consistently achieve strong F1-scores, especially for COPD and ILD, with relatively balanced precision and recall. Overall, deeper MLP models provide better performance across most metrics, particularly for ILD and PN, while SVM models exhibit strong precision but higher FNR for TB and AST. Note that the results for the K-nearest neighbors model, linear discriminant model, ensemble method are again omitted due to their mediocre performance.

**Table 4:** Performance evaluation of various machine learning (ML) and multi-layer perceptron (MLP) models

The SVM (Quadratic) model						
Class	Precision	Recall	F1-score	TNR	FPR	FNR
AST	0.949	0.834	0.888	0.990	0.010	0.166
COPD	0.955	0.807	0.874	0.988	0.012	0.193
ILD	0.693	0.950	0.800	0.914	0.086	0.050
PN	0.860	0.841	0.850	0.968	0.032	0.159
TB	0.915	0.825	0.868	0.981	0.019	0.175
*N*	0.867	0.890	0.878	0.966	0.034	0.110

The SVM (Cubic) model						
AST	0.867	0.841	0.854	0.976	0.024	0.159
COPD	0.853	0.815	0.833	0.977	0.023	0.185
ILD	0.889	0.889	0.889	0.966	0.034	0.111
PN	0.835	0.835	0.835	0.958	0.042	0.165
TB	0.814	0.814	0.814	0.974	0.026	0.186
*N*	0.888	0.888	0.888	0.960	0.040	0.112

The MLP (Narrow) model						
AST	0.925	0.925	0.925	0.990	0.010	0.075
COPD	0.953	0.953	0.953	0.993	0.007	0.047
ILD	0.941	0.941	0.941	0.990	0.010	0.059
PN	0.927	0.927	0.927	0.987	0.013	0.073
TB	0.922	0.922	0.922	0.986	0.014	0.078
*N*	0.926	0.926	0.926	0.985	0.015	0.074

The MLP (Medium) model						
AST	0.968	0.968	0.968	0.996	0.004	0.032
COPD	0.967	0.967	0.967	0.996	0.004	0.033
ILD	0.966	0.966	0.966	0.996	0.004	0.034
PN	0.957	0.957	0.957	0.995	0.005	0.043
TB	0.947	0.947	0.947	0.993	0.007	0.053
*N*	0.946	0.946	0.946	0.993	0.007	0.054

The MLP (Wide) model						
AST	0.963	0.958	0.960	0.992	0.008	0.042
COPD	0.973	0.958	0.966	0.994	0.006	0.042
ILD	0.975	0.975	0.975	0.996	0.004	0.025
PN	0.965	0.958	0.962	0.993	0.007	0.042
TB	0.943	0.927	0.935	0.987	0.013	0.073
*N*	0.952	0.946	0.949	0.990	0.010	0.054

The MLP (Bilayered) model						
AST	0.939	0.929	0.934	0.987	0.013	0.071
COPD	0.932	0.936	0.934	0.986	0.014	0.064
ILD	0.932	0.922	0.927	0.986	0.014	0.078
PN	0.918	0.912	0.915	0.983	0.017	0.088
TB	0.923	0.918	0.921	0.984	0.016	0.082
*N*	0.936	0.932	0.934	0.987	0.013	0.068

The MLP (Trilayered) model						
AST	0.914	0.907	0.911	0.982	0.018	0.093
COPD	0.896	0.902	0.899	0.977	0.023	0.098
ILD	0.893	0.893	0.893	0.977	0.023	0.107
PN	0.876	0.869	0.872	0.973	0.027	0.131
TB	0.874	0.866	0.870	0.973	0.027	0.134
*N*	0.897	0.894	0.896	0.978	0.022	0.106

Table 5 provides a comprehensive performance comparison of various ML and MLP classifiers based on their confusion matrices. We make the following observations. SVM models (quadratic and cubic) perform well on COPD and PN but struggle with ILD and TB, misclassifying them at higher rates. MLP models, especially Wide and Medium, achieve the highest classification accuracy across all classes, particularly for COPD, ILD, and PN, showing minimal confusion. The MLP (Narrow) model has lower accuracy for TB and N, indicating difficulty distinguishing them. Overall, deeper MLP models (Wide, Bilayered, and Trilayered) offer better performance, but the Trilayered model slightly misclassifies TB and Normal cases more frequently than other MLP variants. Finally, the MLP models also excel at detecting the normal class by achieving an accuracy of around 90%, while SVM models are less efficient at differentiating between healthy individuals and those with pulmonary diseases.

**Table 5:** Confusion matrices of various machine learning (ML) and multi-layer perceptron (MLP) models

Algorithms	Actual/Guess	AST	COPD	ILD	PN	TB	N
SVM (Quad.)	AST	**83.4**	1.1	9.1	2.9	1.5	2.0
	COPD	0.9	**80.6**	8.9	2.7	2.9	3.9
	ILD	0.8	0.1	**95.0**	2.1	0.3	1.7
	PN	1.4	1.0	9.1	**84.1**	2.0	2.4
	TB	0.9	1.1	8.8	3.1	**82.4**	3.6
	N	0.5	0.5	6.1	2.9	1.0	**88.9**
SVM (Cubic)	AST	**84.1**	2.0	3.4	5.0	2.1	3.4
	COPD	2.0	**81.5**	2.6	5.9	3.9	4.1
	ILD	2.8	0.3	**88.9**	6.2	0.9	0.8
	PN	3.4	1.4	5.2	**83.5**	2.2	4.4
	TB	2.3	2.0	4.0	6.4	**81.4**	3.9
	N	1.3	0.2	3.5	5.4	0.7	**88.8**
MLP (Narrow)	AST	**92.5**	1.9	0.5	1.1	2.7	1.3
	COPD	0.7	**95.3**	0.8	0.5	1.3	1.3
	ILD	0.7	0.4	**94.1**	1.1	1.6	2.1
	PN	1.3	0.9	1.3	**92.7**	1.7	2.0
	TB	1.2	1.9	1.3	1.2	**92.2**	2.1
	N	0.9	1.2	1.9	1.5	2.0	**92.6**
MLP (Medium)	AST	**96.8**	0.8	0.5	0.2	1.1	0.5
	COPD	0.4	**96.7**	0.7	0.1	1.0	1.1
	ILD	0.2	0.6	**96.6**	0.5	1.1	0.9
	PN	0.6	0.6	0.8	**95.7**	1.1	1.2
	TB	1.3	1.0	0.8	1.0	**94.7**	1.2
	N	0.5	0.8	0.9	1.1	2.0	**94.6**
MLP (Wide)	AST	**95.7**	0.5	0.6	0.7	0.8	1.6
	COPD	0.7	**95.8**	0.3	0.2	1.6	1.4
	ILD	0.4	0.4	**97.5**	0.2	0.6	0.9
	PN	0.7	0.3	0.5	**95.8**	1.3	1.3
	TB	0.7	1.6	1.1	0.8	**92.7**	3.1
	TB	0.7	1.6	1.1	0.8	92.7	3.1
MLP (Bilayered)	AST	92.9	1.3	0.8	0.6	2.3	2.0
	COPD	1.0	93.6	0.3	1.6	1.3	2.1
	ILD	1.5	1.2	**92.2**	1.3	2.0	1.8
	PN	2.1	2.9	1.2	**91.2**	1.5	1.1
	TB	3.1	1.2	1.2	1.2	**91.8**	1.5
	N	1.6	1.4	0.9	0.9	2.0	**93.2**
MLP (Trilayered)	AST	**90.7**	1.6	2.4	1.6	2.8	0.9
	ILD	3.3	1.6	**89.3**	1.7	2.5	1.6
	PN	3.2	2.3	3.6	**86.9**	3.0	1.1
	TB	3.3	2.7	2.3	3.0	**86.6**	2.1
	N	2.0	1.9	1.7	1.6	3.4	**89.4**

The diagonal values (in bold) represent the number of correctly classified instances for each class.

*AST* Asthma, *N* Normal.

**Fig. 3:**
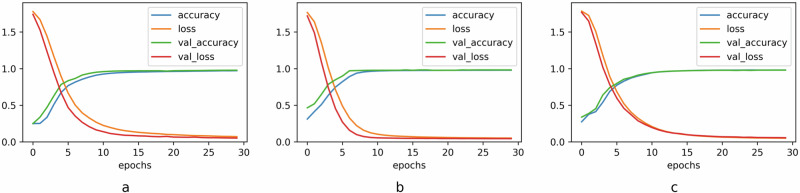
Accuracy and loss functions against the number of epochs, for the three deep learning (DL) models. **a** Vanilla convolutional neural network (CNN) model. **b** Vanilla long short-term memory (LSTM) model. **c** Vanilla transformer.

Figure 3 plots the accuracy and categorical focal cross-entropy loss function against number of epochs during the training and validation phase, for each of the three DL models, i.e., Transformer, LSTM, and CNN. We note that both the loss function and accuracy of the CNN, LSTM, and Transformer classifiers saturate to nearly optimal values in roughly 30 epochs. Figure 3 also clearly illustrates that none of the three DL models exhibits any kind of overfitting or underfitting.

**Fig. 4:**
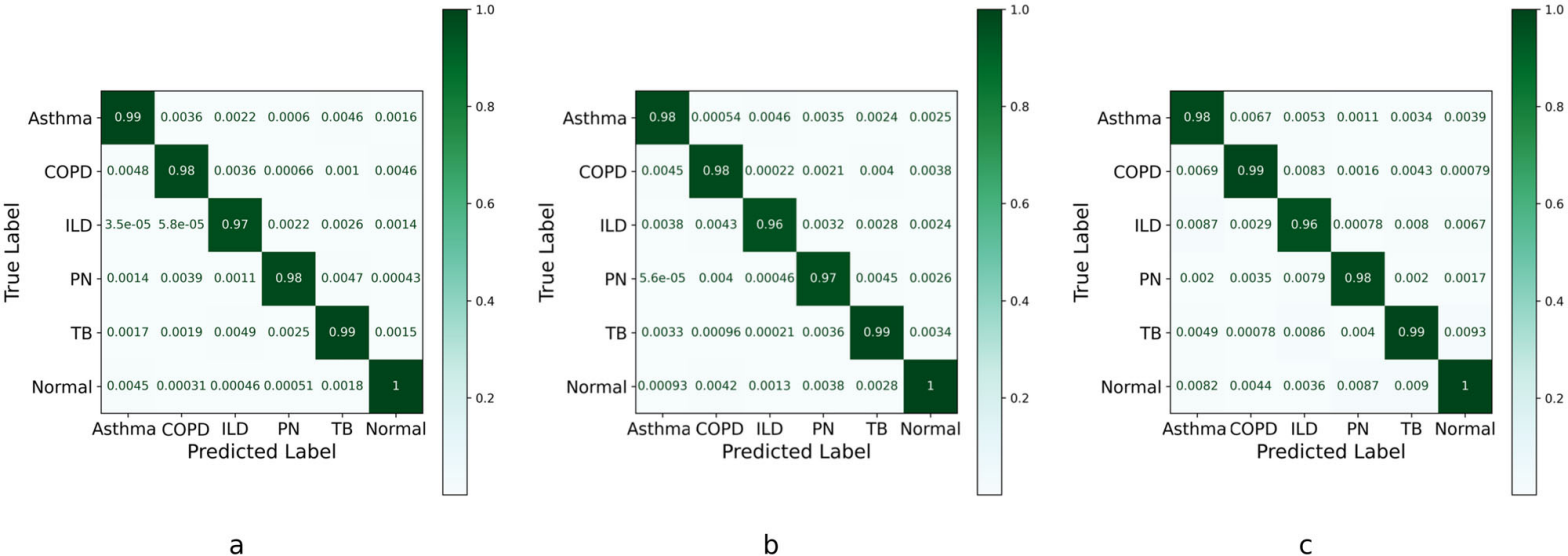
Confusion matrices for the three deep learning (DL) models. **a** Vanilla convolutional neural network (CNN). **b** Vanilla long short-term memory (LSTM). **c** Vanilla transformer.

Next, Fig. 4 plots the confusion matrices of the Transformer, LSTM, and CNN models. We observe the following. (1) All three confusion matrices are nearly diagonal matrices, i.e., diagonal-heavy, which implies that all three DL models achieve very high test precision and recall values. Specifically, the Transformer, LSTM, CNN models achieve an overall recall of 98%, 98%, and 99%, respectively. (2) All three DL models report a recall of 100% for the Normal class. This is a pleasant result because the respiratory performance of a healthy person is quite distinct from that patients with pulmonary diseases. (3) The CNN model slightly outperforms the other two DL models in diagnosing Asthma with a recall of 99%. (4) All three DL models report slightly less recall of (96–97)% for the ILD class.

Table 6 extends further the discussion of Fig. 4 by providing a comprehensive performance comparison of the three DL models in terms of accuracy, precision, recall, F1-score, support, and many other metrics. Firstly, Table 6 re-affirms the fact that the normal breathing pattern is quite distinct compared to pathological respiratory patterns due to Asthma, COPD, ILD, PN, and TB. Secondly, the CNN, LSTM, and Transformer models have the largest precision of 1 for the ILD and PN classes, ILD class, Asthma class, respectively. This implies that the CNN, LSTM, and Transformer models never misdiagnose a healthy person (or a person with any other pulmonary disease) with having ILD and PN, ILD, Asthma, respectively. Thirdly, the precision (recall) of the Transformer model for the COPD class is the lowest (highest) among all five diseases, i.e., 0.94 (0.99), while the CNN has a high precision of 0.99 and high recall of 0.98 for the COPD class. This implies that the Transformer model is biased towards the minimization of the false negatives for the COPD class. Fourthly, we note that the Transformer model has a slightly lower F1-score of 96%, respectively, for the COPD class, which makes it slightly less reliable for diagnosing the COPD disease. On the other hand, the CNN registers a very high F1-score of ≥98% for all five disease classes. In terms of TNR, FPR, and FNR metrics, all three models achieve high performance, with minimal false positives and false negatives. As for the MCC and Jaccard index, LSTM registers higher MCC and Jaccard Index values, especially for ILD, when compared to the Transformer model and CNN. Overall, the CNN model turns out to be the winner as it strikes a good balance between precision and recall, which in turn makes it suitable for lung disease diagnosis in a clinical setting where both false positives and false negatives have great implications.

**Table 6:** Performance evaluation of convolutional neural network (CNN), long short-term memory (LSTM), and Transformer models (with estimated per-class accuracy)

The CNN model										
Class	Accuracy	Precision	Recall	F1-score	TNR	FPR	FNR	MCC	Jaccard index	Support
Asthma	0.99	0.98	0.99	0.98	1.00	0.00	0.02	0.98	0.97	23520
COPD	0.99	0.99	0.98	0.98	1.00	0.00	0.02	0.97	0.95	28224
ILD	0.98	1.00	0.97	0.99	1.00	0.00	0.03	0.97	0.96	27048
PN	0.99	1.00	0.98	0.99	1.00	0.00	0.03	0.97	0.95	19992
TB	0.99	0.98	0.99	0.98	0.99	0.01	0.02	0.97	0.96	16464
Normal	1.00	1.00	1.00	1.00	1.00	0.00	0.01	0.98	0.97	27048

The LSTM model										
Asthma	0.99	0.99	0.98	0.99	1.00	0.00	0.01	0.98	0.97	23520
COPD	0.99	0.95	0.98	0.97	1.00	0.00	0.01	0.98	0.97	28224
ILD	0.98	1.00	0.96	0.98	1.00	0.00	0.02	0.99	0.98	27048
PN	0.98	0.99	0.97	0.98	1.00	0.00	0.01	0.98	0.97	19992
TB	0.99	0.99	0.99	0.99	1.00	0.00	0.01	0.98	0.97	16464
Normal	1.00	0.99	1.00	1.00	1.00	0.00	0.01	0.98	0.97	27048

The transformer model										
Asthma	0.98	1.00	0.98	0.99	0.99	0.01	0.02	0.97	0.95	23520
COPD	0.99	0.94	0.99	0.96	1.00	0.00	0.02	0.98	0.96	28224
ILD	0.97	0.99	0.96	0.97	0.99	0.01	0.03	0.96	0.94	27048
PN	0.99	0.99	0.98	0.98	1.00	0.00	0.02	0.98	0.97	19992
TB	0.99	0.99	0.99	0.99	0.99	0.01	0.03	0.97	0.95	16464
Normal	1.00	1.00	1.00	1.00	1.00	0.00	0.03	0.97	0.95	27048

### Ablation study

**Fig. 5:**
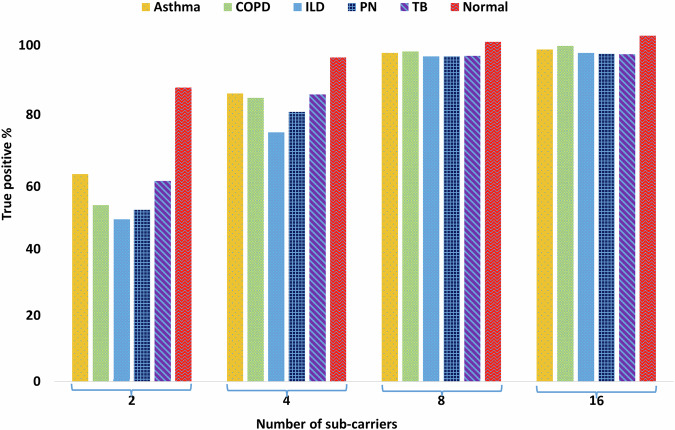
The Ablation study. This study assesses the impact of the number of sub-carriers on performance of the Transformer classifier.

Next, we systematically and monotonically decrease the number of sub-carriers from 16 to 2, in order to quantify how this critical design parameter impacts the performance of our Transformer model. Figure 5 reveals that the Transformer model needs to radio-observe the human chest on at least 8 different microwave frequencies, in order to make a reliable diagnosis (with 96% accuracy) of the underlying lung disease. Thus, Fig. 5 serves as an important benchmark for the 6G ISAC health sensing systems of future that intend to do information exchange and health sensing simultaneously. Precisely speaking, Fig. 5 illustrates that the minimum sensing overhead required by our Transformer model to achieve an accuracy of 96% is 8/64 = 12.5% of the allocated bandwidth, which is consumed by ISAC on need basis only. This is quite remarkable because high performance of opportunistic ISAC with very few sub-carriers implies that we have more bandwidth (and thus more data rate) for information exchange.

We note that OFDM has some drawbacks, such as a high peak-to-average power ratio, sensitivity to frequency offset and phase noise leading to inter-carrier interference, and the need for a cyclic prefix, which reduce the transmission rates. However, these limitations primarily impact communication applications such as 6G, whereas this work focuses on using OFDM for lung disease diagnosis. We chose OFDM because its multi-carrier nature enables a wide frequency response analysis of the human chest, akin to radio spectroscopy. This is supported by our ablation study (see Fig. 5), which demonstrates that we need to radio-observe a person on at least eight frequencies for reliable lung disease diagnosis. In contrast, single-carrier schemes like phase shift keying and quadrature amplitude modulation lack the sensitivity to distinguish between different pulmonary diseases.

### Performance comparison with the state-of-the-art(SOTA)

Traditionally, lung disease classification has relied on clinical evaluations performed by medical professionals, often supported by diagnostic tools such as chest X-rays, CT scans, and auscultation via stethoscopes. With the advancement of machine and deep learning techniques, automated classification using medical imaging and audio signals has gained substantial momentum. Although imaging-based approaches, such as chest X-rays and CT scans, have demonstrated strong classification performance, they also present several limitations, including exposure to ionizing radiation, high operational costs, dependence on trained medical personnel, and the requirement for specialized clinical infrastructure. Similarly, methods that combine ML/DL with lung sounds recorded via stethoscopes and cough audio have shown promising results. However, following the COVID-19 pandemic, there has been a significant shift in research focus toward fully non-contact and non-invasive diagnostic solutions, driven by increased safety concerns and the growing demand for scalable, remote healthcare systems. This shift has paved the way for the development of radar-based and SDR-based techniques that utilize radio frequency signals for respiratory monitoring. However, many early RF-based studies were constrained by small datasets and often relied on healthy volunteers simulating abnormal respiratory patterns, thus limiting their clinical applicability. In contrast, our proposed method leverages a significantly larger and more diverse real-time dataset collected from clinically diagnosed patients with respiratory diseases, enabling a more accurate, robust, and practically relevant evaluation of non-contact respiratory disease classification using RF technologies. Furthermore, we employ a SDR system operating at a frequency of 5.23 GHz, which lies within the non-ionizing electromagnetic spectrum. This ensures safe, non-invasive monitoring suitable for continuous health assessment. A detailed comparison with existing state-of-the-art methods is provided in Table 7, highlighting the advantages and contributions of our approach.

**Table 7:** Comparison of proposed 6G/WiFi integrated sensing and communication (ISAC) passive sensing-based contactless pulmonary disease screening method with state-of-the-art techniques

Reference	Sensing/ dataset used	Model	Lung/ respiratory diseases identified	Participants	Performance	Remarks
Malik et. al.^13^	X-ray images	VGG-19, ResNet-50, Inception v3, CDC_Net	COVID-19, Lung Cancer, Pneumothorax, TB, Pneumonia	COVID-19: 2371, Pneumothorax: 12,000, Pneumonia: 3867, Lung Cancer: 5000, TB: 4200, Normal: 2949, Total: 30387	VGG-19: 95.61%, ResNet-50: 96.15%, Inception v3: 95.16%, CDC_Net: 99.39%	Proposed CDC-Net, a CNN with residual connections and dilated convolutions to enhance classification accuracy.
Tuncer et. al.^14^	X-ray images	DT, LD, SVM, Ensemble, k-NN	COVID-19, Pneumonia, Normal	COVID-19: 135, Pneumonia: 150, Normal: 150, Total: 435	DT: 86.09%, LD: 82.30%, SVM: 97.01%, k-NN: 96.09%, Ensemble: 93.10%	616 features extracted from X-rays to improve the classification and classified with six ML models.
Mei et. al.^18^	CT scans	CNN	ILD and Normal	ILD: 458	Sensitivity: 82.4%	Classified ILD and predicted progression and prognosis using CT scan data.
Choi et. al.^22^	Respiratory Sound Database	Light Attention, VGGish	Bronchiectasis, COPD, ILD, Pneumonia, Asthma, Normal	Total: 126 (Normal: 12, Asthma: 23, COPD: 20, ILD: 26, Pneumonia: 20, Bronchiectasis: 25)	Overall: 92.81%, Class-wise accuracy between 89 to 97%	Used electronic stethoscope for data collection; introduced LACNN with XAI for interpretability.
Xu et. al.^27^	Cough based Database	MobileNet	COPD, Asthma, Chronic cough, Normal	Total: 201 (Normal: 50, Asthma: 94, COPD: 34, Asthma + COPD: 13, Chronic cough: 10)	94.8	built an end-to-end audio-based cough detection model using a pre-trained MobileNet and a variety of audio augmentation techniques.
Siddiqui et. al.^34^	UWB radar	LSTM, SVM, KNN, Decision Tree	COPD and Healthy	70 (35 COPD, 35 healthy)	LSTM: 96.3%, SVM: 94.5%, KNN: 92.7%, DT: 90.9%	Radar-based contactless system for COPD detection.
Beltrao et. al.^40^	CW radar dataset	Nonlinear Least Squares (NLS) Estimator	Normal and Cheyne-Stokes	12 premature infants	97%	Monitored respiratory patterns of premature infants using radar
Rehman et. al.^45^	SDR based CSI	Cosine KNN, Complex Tree, Boosted Tree, SVM	Eupnea, Bradypnea, Tachypnea, Biot, Sighing, Kussmaul, Cheyne-Stokes, CSA	5 subjects, 8 patterns, 10 repetitions = 400 samples	Cosine KNN: 97.5%, Complex Tree: 96.8%, Boosted Tree: 85.6%, SVM: 75.5%	Non-invasive recognition of 8 abnormal patterns.
Kawish et. al.^46^	SDR based CSI	K-NN, SVM	Eupnea, Tachypnea, Bradypnea	4 subjects, 3 breathing pattern, 5 repetitions = 60 experiments total	Chest based performance: 95.9%, Hand based performance: 88.1%	Monitored breathing patterns using SDR-based CSI reflected from the chest, and also attempted to determine these patterns from reflections off the hand.
Proposed method	OFDM-based SDR data	MLP (Medium)	Five lung diseases + Healthy	190 + 30	95.9%	Explained in section III-B
Proposed method	OFDM-based SDR data	CNN	Five lung diseases + Healthy	190 + 30	98%	Explained in section III-B

## Discussion

Under the proposed method, the transmit SDR exposes the chest of the subject to the OFDM signal, while the receive SDR collects the signal reflected off the chest of the subject. This lets us acquire our custom dataset—the so-called *OFDM-Breathe dataset*, which we believe is first of its kind.

To the best of our knowledge, the OFDM-Breathe dataset is the *first* labeled dataset that captures *five kinds of respiratory disorders* (i.e., *Asthma, COPD, ILD, PN, TB*) *in actual patients* via OFDM signals in the microwave band. Also, OFDM-Breathe dataset is the *largest* among all previously reported datasets that utilize OFDM signals in the microwave band for various health sensing tasks. Specifically, OFDM-breathe dataset consists of 26,760 s of raw RF data (at 64 distinct OFDM frequencies in the microwave band) that we have acquired from a total of 220 subjects (30 healthy control group, and 190 patients diagnosed with five kinds of respiratory diseases). Finally, OFDM-Breathe dataset is collected in the city of Lahore, Pakistan during the smog season (i.e., from October 2023 to January 2024, and during January 2025). The city of Lahore consistently remained among one of the most polluted cities in the world during the winter season of the years 2023-25 in terms of PM2.5, PM10 concentration, and air quality index^57^. Thus, the *OFDM-Breathe dataset could provide valuable insights into the aggregate impact of extreme environment (pollution) on respiratory health of the civic population of Lahore*.

**Fig. 6:**
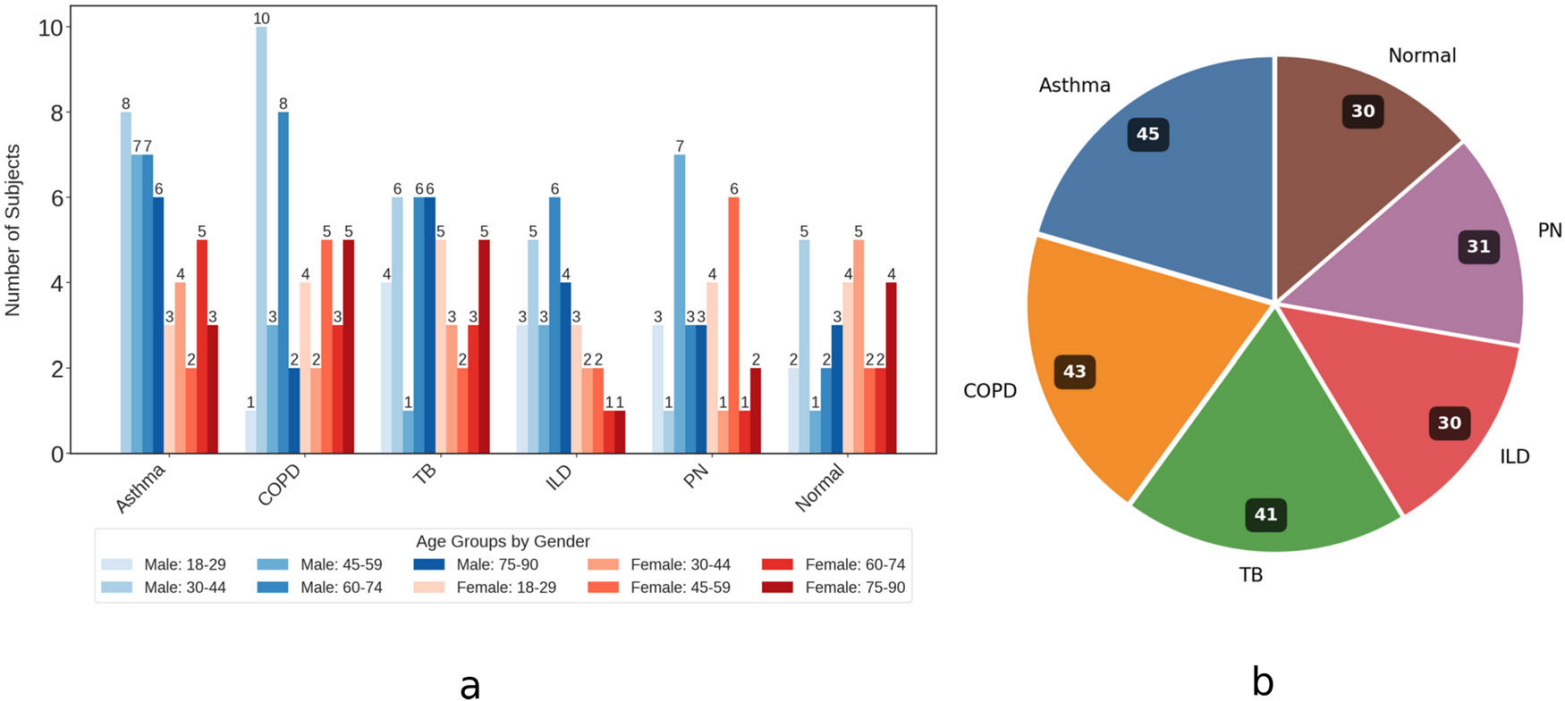
Statistical overview of the orthogonal frequency division multiplexing (OFDM)-breathe dataset: disease class distribution and demographic age distribution by gender. **a** Age distribution of subjects by disease and gender. **b** Distribution of disease and healthy control classes.

Following comments about the OFDM-Breathe dataset are in order. (i) This is a cross-sectional study whereby the dataset consists of 125 male subjects with an average age of 54.2 years, and 95 female subjects with an average age of 48.6 years. More detailed breakdown of the age distribution of the subjects (male, female) with respect to the five diseases is provided in Fig. 6a. (ii) The dataset comprises 30 subjects from the healthy control group, with the remaining 190 subjects being formally diagnosed patients experiencing an active onset of one of the following respiratory diseases: (1) Asthma, (2) COPD, (3) TB, (4) ILD, and 5) PN. (iii) The data of the healthy control group is collected from healthy subjects (students and staff) at university main campus. (iv) The data of the lung disease patients is collected from a local hospital in Lahore. v) For each of the five lung diseases, the labels are assigned by the on-duty clinician (who diagnosed the disease using clinical symptoms and chest X-rays). Further, data is collected from those admitted patients only who were stable and conscious. (vi) Among the 190 patients diagnosed with a respiratory disease, 45 have Asthma, 43 have COPD, 41 have TB, 30 have ILD, and 31 have PN (see Fig. 6b). Note that the OFDM-Breathe dataset is slightly imbalanced, from the perspective of ILD and PN patients. Finally, in addition to the radio data, we also collected the following metadata from the subjects: Hypertension (yes/no), family history of respiratory or chronic disease (yes/no), Diabetes Mellitus (yes/no), smoking (yes/no), low Oxygen saturation (if SpO2 < 95%), elevated Temperature (if  >98.6 F), elevated respiratory rate (if  >20 breaths/min), and high pulse (if  >100 bpm).

The Data collection protocol for OFDM-Breathe dataset is as follows. Under proposed method for data collection, each participant sits on a chair that is roughly 2.5 feet away from a table that hosts the two USRP SDRs and the two directional horn antennas which both point towards the chest of the subject (see Fig. 1). The transmit antenna strikes an OFDM signal onto the chest of the subject, while the receive antenna collects the signals reflected off the subject’s chest. For the duration of experiment (which is 30 s), the subject remains stationary in order to make sure that there are no motion artifacts in the collected data. For each subject, we repeat the experiment four times. This allows us to collect 90 min of data for Asthma, 86 min of data for COPD, 82 min of data for TB, and 60 min of data for normal classes, 60 min of data for the ILD class, and 62 min of data for the PN class. Overall, the OFDM-Breathe dataset consists of a total of 440 min of raw CFR data, due to a total of 880 (=220 × 4) experiments.

## Conclusion

To sum things up, this work investigated a non-contact method whereby the pulmonary patients were radio-exposed with non-ionizing 6G/WiFi microwave OFDM signals in order to screen for five respiratory diseases, i.e., Asthma, COPD, ILD, PN, and TB. We acquired OFDM-Breathe dataset, first of its kind, which consists of 26,760 s of raw RF data at 64 distinct microwave OFDM frequencies, acquired from a total of 220 subjects in a hospital setting (30 healthy control subjects, and 190 pulmonary patients). Among the 190 patients, 45 have Asthma, 43 have COPD, 41 have TB, 30 have ILD, and 31 have PN. We implemented a number of ML and DL models in order to do lung disease classification using OFDM-Breathe dataset. Among all AI models, the CNN model turned out to be the winner in terms of precision, recall, and F1-score. The ablation study revealed that our proposed microwave-based spectroscopy method needs to radio-observe human chest on eight different microwave frequencies only, in order to make a reliable diagnosis of the underlying lung disease. This corresponds to a small sensing overhead of 12.5%, which points to the feasibility of 6G ISAC systems of future.

This work is primarily a feasibility study whereby we collected radio data from a cohort of 190 lung disease patients at a local hospital, and utilized random splitting method to evaluate the performance of various DL models for lung disease classification. However, evaluating the performance of the DL models by doing individual-dependent testing on unseen data in order to assess their generalization capability is crucial^58^. To this end, we aim to pursue clinical trials involving a larger patient cohort to validate, fine-tune, and evaluate our DL models at the subject-level on the unseen data. Upon successful completion of clinical trials, we will initiate the regulatory approval process in line with local and international standards, ensuring our SDR-based diagnostic system complies with safety and efficacy requirements for medical devices. Additionally, we plan to perform a comprehensive cost-benefit analysis, emphasizing the system’s affordability, portability, and compatibility with existing medical infrastructure. The fact that the proposed method is built upon non-ionizing commodity 5G/WiFi signals makes it a viable add-on to current healthcare setups, offering diagnostics without disrupting existing clinical workflows. The proposed method’s potential for integration with smart radio devices (e.g., smartphones, routers, IoT boards) makes it cost-effective and aligns with global trends toward AI-powered, non-contact, patient-centric healthcare, which is especially relevant in post-COVID-19 smart cities and telemedicine. In Pakistan and other resource-constrained regions in the third world, this low-cost, scalable solution can bridge healthcare access gaps, benefiting both urban and rural communities. To this end, we aim to engage with clinicians, pulmonologists, policymakers, and technology experts to pilot, refine, and prepare the proposed system for real-world deployment in the coming years.

## Supplementary information

Description of Additional Supplementary Files

Dataset 1

Dataset 2

Dataset 3

Dataset 4

Reporting Summary

## Data Availability

The source data for Figs. 6–5 of this manuscript are available as datasets 1–4 (i.e., Fig. 3.xlsx, Fig. 4.xlsx, Fig.5.xlsx, Fig. 6.xlsx).

## Code availability

The ML and MLP-based models were trained and evaluated using Matlab’s classification learner app. The DL based models were trained and evaluated using TensorFlow framework in Python. The Python codes for the DL models are available from corresponding author on a reasonable request.
