# Supplementary material for: Non-contact lung disease classification via orthogonal frequency division multiplexing-based passive 6G integrated sensing and communication
Source: Commun Med (Lond). 2026 Jan 6;6:9. doi: 10.1038/s43856-025-01181-2 (PMC12774925; doi:10.1038/s43856-025-01181-2)
Supplement: Supplementary file 1 — Description of Additional Supplementary Files [file 43856_2025_1181_MOESM1_ESM.docx]

**Description of Additional Supplementary Files**

Four supplementary files are attached to this submission: Fig3.xlsx, Fig4.xlsx, Fig5.xlsx, Fig6.xlsx.

**Dataset 1 (Fig3.xlsx)**

**Description:**

Dataset 1 contains the source file Fig3.xlsx which contains the source data for Fig. 3 of the manuscript.

**Dataset 2 (Fig4.xlsx)**

**Description:**

Dataset 2 contains the source file Fig4.xlsx which contains the source data for Fig. 4 of the manuscript.

**Dataset 3 (Fig5.xlsx)**

**Description:**

Dataset 3 contains the source file Fig5.xlsx which contains the source data for Fig. 5 of the manuscript.

**Dataset 4 (Fig6.xlsx)**

**Description:**

Dataset 4 contains the source file Fig6.xlsx which contains the source data for Fig. 6 of the manuscript.
